# Astrocytes from lamina cribrosa are involved in the autoregulatory function of optic nerve head vessels *in vitro*

**DOI:** 10.3389/fmolb.2025.1636882

**Published:** 2025-08-13

**Authors:** Xiaoxiao Feng, Wenjia Zhang, Tingting Wan, Kangwei Jiao, Liwei Zhang, Changhui Li, Libo Xiao

**Affiliations:** ^1^ Department of Ophthalmology, Affiliated Hospital of Yunnan University, Yunnan, China; ^2^ Yunnan Ophthalmic Disease Clinical Medical Center, Yunnan, China

**Keywords:** oxygen-glucose deprivation/reperfusion, lamina cribrosa astrocytes, vascular smooth muscle cells, mTOR pathway, optic nerve head (ONH)

## Abstract

**Purpose:**

To investigate the role of the lamina cribrosa (LC) astrocytes in the autoregulatory capacity of optic nerve head (ONH) vessels and to explore the underlying molecular mechanisms.

**Methods:**

The Oxygen-glucose deprivation/reperfusion model (OGD/R) *in vitro* was constructed to examine the changes in cell morphology and protein expression in LC astrocytes. LC astrocytes were co-cultured with vascular smooth muscle cells (VSMCs) to detect the role of LC astrocytes in the autoregulatory function of vessels.

**Results:**

The partial pressure of oxygen (PO_2_) in the supernatant of LC astrocytes was significantly lower following OGD, and this reduction was more pronounced with longer OGD durations. OGD inhibited proliferation and promoted apoptosis in LC astrocytes, with longer OGD durations correlating with decreased proliferation and increased apoptosis. Reoxygenation following 1 h of OGD led to upregulation of GFAP, mTOR, cPLA_2_ protein expression and supernatant PGE2 concentration in LC astrocytes, an effect that can be attenuated by the mTOR inhibitor. Co-culturing with LC astrocyte resulted in increased expression of MYPT1 protein in VSMCs, and the VSMCs exhibited a relaxed morphology.

**Conclusion:**

Under *in vitro* OGD/R conditions, LC astrocyte were activated through the mTOR pathway, leading to increased secretion of PGE_2_, which locally regulates the dilation of VSMCs. In conclusion, LC astrocytes may regulate local blood flow in the ONH.

## Introduction

Local blood flow stability is essential for maintaining normal function of the optic nerve and retina. When the ocular arterial pressure fluctuates, the vessels of the optic nerve head (ONH) can autoregulate blood flow without control from the autonomic nervous system, thereby maintaining the metabolic needs of visual cells. This phenomenon is recognized as the autoregulatory behavior of the vascular beds (Arnold, 2023). During reactive hyperemia, ONH vessels provide sufficient oxygen and nutrition to prevent regional neuronal ischemic injury in the optic nerve or retina. If the autoregulatory ability is unregulated or defective, pathological changes in regional optic nerve fiber cells lead to visual impairment, which is considered as one of the possible causes of glaucoma or ischemic optic neuropathy (Safa et al., 2022). Thus, a clearer understanding of the cellular and molecular mechanisms underlying the autoregulatory ability of ONH vessels could be contribute to understanding the etiology and exploring treatment options for eye diseases related to optic nerve fiber ischemic injury.

Astrocytes are central integrators and executors for ONH vessels to perform autoregulatory function (Wen et al., 2023; Vecino et al., 2016; Puebla et al., 2022; Paisley and Kay, 2021). In physiologic state, astrocytes serve as templates for blood vessel formation to maintain the blood-retinal barrier (BRB) and regulate vascular tone and blood flow. In pathological conditions, activated reactive astrocytes are protective in the early stages, attempting to maintain the BRB, remove toxins, and limit the spread of damage; in the late stages, they release a variety of proinflammatory cytokines, chemokines, and adhesion molecules, leading to BRB disruption, vascular leakage, formation of aberrant neovascularization, amplification of inflammation, and pathological remodeling of the extracellular matrix. Lamina cribrosa (LC) is a critical component of the ONH. As barrier between the inner and outer portions of the posterior eye, the LC participates in maintaining the stability of the intraocular environment (Strickland et al., 2022). The complex lamellar vascular network of the LC directly integrates into the multi-layered mesh of connective tissue bundles to nourish the axons of retinal ganglion cells. Therefore, the LC is considered a fundamental structure in the autoregulatory function of ONH vessels (Tan et al., 2018). Moreover, various functional cells (e.g., astrocytes, LC cells, microglia) populate the mesh or encase, each fulfilling distinct roles (Tang et al., 2022). Among them, LC astrocytes, the predominant functional cells type in the LC, surround the vessels and form an interface with connective tissues. However, in recent years, the understanding of LC astrocytes has been mainly derived from studies focusing on glial scar formation (Liu et al., 2022) or extracellular matrix remodeling (Boal et al., 2021). The cellular mechanisms underlying autoregulatory function of ONH vessels by LC astrocytes remain unknown.

Recently, the mammalian target of serine/threonine-protein kinase mTOR (mTOR) pathway has garnered significant attention, as it is believed to integrate intracellular and extracellular signals, regulate cell growth and proliferation, and stabilize the intercellular environment and extracellular matrix (Liu and Sabatini, 2020; Hua et al., 2019; Murugan, 2019). Additionally, studies have demonstrated that the mTOR pathway is involved in regulating oxygen metabolism across various cell types (Chun and Kim, 2021; Yang et al., 2022). Based on the above, we hypothesize that this pathway may coordinate the relationship between LC astrocytes and the autoregulatory function of vessels. The aim of this study was to assess the role of LC astrocytes in the autoregulatory function of ONH vessels and to investigate the underlying molecular mechanisms.

## Materials and methods

### Primary culture of LC astrocyte

Healthy Specific Pathogen-Free (SPF)-grade Sprague Dawley (SD) rats (6–8 weeks old) of both sexes was purchased from the Laboratory Animal Production Department, Southern Medical University. The rats were housed in separate cages in the barrier area of the Animal Experimental Center, Sun Yat-sen Eye Center, Sun Yat-sen University. The SD rats were acclimatized in a holding room at 23 °C ± 4 °C with relative humidity 50% ± 5% for 7 days prior to surgery. In accordance with the ARRIVE guidelines, every effort was made in this study to minimize the number of animals used and to reduce animal suffering. SD rats with normal anterior segments, ciliary bodies, and vitreous structure, free of abnormal hemorrhage and exudation, were selected for the experiment. Primary culture of LC astrocyte was prepared from the LC of adult SD rats, as described previously (Lopez et al., 2020), with minor modifications. According to the AVMA Guidelines on Euthanasia, SD rats were euthanized by intraperitoneal injection of 200 mg/kg sodium pentobarbital, and the eyes were subsequently used for primary culture of LC astrocytes. Briefly, the LC was dissected from the optic disc under sterile conditions. The tissues were seeded in 12-well plates containing 2 mL of Dulbecco modified eagle medium (DMEM; 11,965,084; Gibco, Grand Island, NY, United States) per well, supplemented with 20% fetal bovine serum (FBS; 10099141C; Gibco, Grand Island, NY, United States) and penicillin/streptomycin. After 10–14 days in culture (37 °C, 5% CO_2_), non-astrocytic cells, such as neurons and LC cells, were removed by changing the medium. The isolated and purified LC astrocytes were cultured in astrocyte growth medium (CM-M188; Pricella, Wuhan, China) containing 5% FBS. Immunofluorescent staining for glial fibrillary acidic protein (GFAP) revealed that >98% of cells were GFAP-positive astrocytes. This study was approved by Ethics Committee of Affiliated Hospital of Yunnan University/the Second People’s Hospital of Yunnan Province (YNUCARE20210007), and was conducted in accordance with the Association for Research in Vision and Ophthalmology (ARVO) guidelines.

### Primary culture of vascular smooth muscle cells (VSMCs)

Primary VSMCs were isolated from the thoracic aorta media of SD rats, as described previously (Bertoni et al., 2020). The thoracic aortic media was identified and isolated under sterile conditions. The tissues were dissociated, and resulting cells were plated into 25-cm^2^ culture flasks containing 4 mL of DMEM (11,965,084; Gibco, Grand Island, NY, United States) supplemented with 20% FBS (10099141C; Gibco, Grand Island, NY, United States). The cells reached confluence in approximately 4 weeks (37 °C, 5% CO_2_). Over 98% of the cells were confirmed as positive for smooth muscle α-actin (α-SMA; ab124964; 1:300, Abcam, Cambridge, MA, United States).

### Oxygen-glucose deprivation/reperfusion (OGD/R)

OGD/R was performed in confluent LC astrocytes cultured *in vitro*. Cells were washed twice with phosphate-buffered saline (PBS), transferred to an oxygen-depleted, glucose-free AGM medium, and incubated in a hypoxic chamber (94% N2, 5% CO2, 1% O2, 37 °C) for the indicated durations (15 min, 30 min, 1 h, 2 h, 4 h, and 8 h). The cell culture chamber for hyperoxia was strictly regulated by an C21 oxygen and carbon dioxide controller (BioSpherix, Lacona, NY, United States). At the end of the OGD, the cultures were returned to standard medium and incubated under normal conditions (5% CO_2_/95% air, 37 °C) for 12, 24, and 72 h, or 1 week, for reperfusion.

### LC astrocytes-VSMCs co-culture experiments

Non-contact co-culture of LC astrocytes and VSMCs was performed using Transwell, with LC astrocytes inoculated in the lower chamber and VSMCs in the upper chamber. Briefly, LC astrocytes were seeded to confluence on the bottom of 60-mm Transwell. Primary VSMCs were plated on 24-mm chamber in 6-well plates at a density of 100,000 cells per well. At the time of OGD/R, the chamber with VSMCs were placed in the Transwell containing the confluent monolayer of LC astrocytes for 12, 24, or 72 h, or for 1 week. After OGD/R, the chamber with VSMCs were removed and processed for immunofluorescence, while the cell lysates of LC astrocytes were collected for Western blot analysis.

### mTOR inhibitor experiments

For inhibitor studies, 100 nm/mL rapamycin (S1039; Selleck, Houston, TX, United States), an mTOR inhibitor (Querfurth and Lee, 2021), was incubated with LC astrocyte for 1 h prior to the addition of OGD/R stimuli, as described previously (Zha et al., 2021). In the Con groups, 0.01% DMSO was used instead of the rapamycin to pre-treated LC astrocyte cultures before the OGD/R exposure. Cell morphogenesis and protein expression were observed.

### Cell proliferation and apoptosis assay

Cell viability following various OGD treatments was measured using the Cell Counting Kit-8 assay (CCK-8; CK04; Dojindo, Kumamoto, Japan), according to the manufacturer’s protocol. Briefly, cells in 96-well plates (AM-2 ML-RD-S; Corning, Corning, NY, United States) were cultured under OGD for different durations. A 10-μL solution of CCK-8 solution was added to each well, and cells were incubated in a humidified incubator (37 °C, 5% CO2) for 2 h. The optical density at 450 nm was measured using a xMark microplate reader (Bio-Rad, Hercules, CA, United States).

To detect cell apoptosis, terminal deoxynucleotide transferase-mediated dUTP nick-end labeling (TUNEL; C10245; Thermo Fisher Scientific, Waltham, MA, United States) staining was performed, following the manufacturer’s protocol. After OGD treatment on coverslips, cells were fixed in 4% paraformaldehyde and permeabilized with 0.1% Triton X-100. TUNEL reaction buffer was added to each coverslip and incubated for 60 min at 37 °C in the dark. Coverslips were counterstained with DAPI. Coverslips were rinsed and mounted on glass microscope slides (Vector Laboratories, Burlingame, CA, United States), and apoptotic cells were imaged and counted in three different fields on each coverslip using BX53 fluorescence microscopy (Olympus, Tokyo, Japan).

### Partial pressure of oxygen (PO_2_) monitoring in cellular supernatant

To confirm the hypoxia or normoxia status in cells during OGD/R, an Oxylite oxygen-sensitive microelectrode probe (Oxford Optronix, Rochester Hills, MI, United States) was used to monitor the oxygen concentration in the cell supernatant, following the manufacturer’s instructions. The sterile oxygen-sensitive probe was immersed at least 2 mm below the liquid surface of the cell supernatant to measure and record the oxygen concentration. Recordings were taken for 1 min until the data stabilized, ensuring that the deviation remained below 10%. Four replicates were measured per group. A PO_2_ value below 10 mmHg under hypoxic condition confirmed successful OGD.

### Enzyme-linked immunosorbent (ELISA) assay

The cell culture medium was collected following the OGD/R treatment. The release of prostaglandin E2 (PGE_2_) from the cell supernatant was measured in duplicate using ELISA (PKGE004B; R&D Systems, Minneapolis, MN, United States) according to the manufacturer’s instructions. In brief, the calibration diluent (150 μL) was added to each well of the microplate, followed by the addition of the standard substance (150 μL) and the cell supernatant sample (150 μL), respectively. Afterwards, the primary antibody (50 μL) and the PGE2 conjugate (50 μL) were added to each well in sequence, and incubated at room temperature for 1 h and 2 h, respectively. After washing the microplate four times, substrate solution (200 μL) was added and incubated at room temperature for 30 min, followed by the addition of stop solution (100 μL). The absorbance was measured at a wavelength of 450 nm using a microplate reader (A51119700C; Thermo Fisher Scientific). The level of PGE2 in the sample was calculated based on the standard curve.

### Immunofluorescence microscopy

Cells grown on glass coverslips were washed twice with PBS, fixed with 4% paraformaldehyde for 15 min, permeabilized with 0.1% Triton X-100 for 10 min, blocked with 5% FBS for 30 min at 37 °C, and then incubated at 4 °C for 24 h with following primary antibodies: rabbit polyclonal antibody to glial fibrillary acid protein (GFAP; ab7260; 1:5,000; Abcam, Cambridge, MA, United States), rabbit polyclonal antibody to cytosolic phospholipase A2 (cPLA2; ab73406; 1:500; Abcam, Cambridge, MA, United States), rabbit monoclonal antibody to mTOR (2,983; 1:200; Cell Signaling Technology, Danvers, MA, United States), rabbit monoclonal antibody to phosphorylated mTOR (p-mTOR; 5,536; 1:100; Ser2448; Cell Signaling Technology, Danvers, MA, United States), and rabbit polyclonal antibody to myosin phosphatase target subunit 1 (MYPT1; ab59235; 1:100; Abcam, Cambridge, MA, United States), or PBS (as negative control). After washing with PBS, cells were incubated at 37 °C for 1 h with the following secondary antibodies: goat anti-rabbit IgG H&L (ab150077; 1:500; Alexa Fluor® 488; Abcam, Cambridge, MA, United States). The cell nuclei were stained with DAPI, and fluorescent images were acquired using a Microphot-FX Research Microscope (Nikon, Tokyo, Japan). Five fields of view were randomly selected from each biological replicate. The overall mean fluorescence intensity of each field of view was analyzed using ImageJ software. The average value of the five fields of view represents the mean fluorescence intensity of this biological replicate.

### Western blotting

Proteins were extracted from rat cells using a protein extraction kit (P0013G; Beyotime, Beijing, China). Protein concentrations were quantified using a BCA protein assay kit (23,225; Pierce, Rockford, IL, United States), and proteins were separated by 8%–12% SDS-PAGE electrophoresis, then transferred to PVDF membranes (1,620,177; Bio-Rad, Hercules, CA, United States). Membranes were incubated with the following primary antibodies: rabbit polyclonal antibody to GFAP (ab7260; 1:5,000; Abcam, Cambridge, MA, United States), rabbit polyclonal antibody to cPLA2 (ab73406; 1:1,000; Abcam, Cambridge, MA, United States), rabbit monoclonal antibody to mTOR (2,983; 1:1,000; Cell Signaling Technology, Danvers, MA, United States), rabbit monoclonal antibody to p-mTOR (5,536; 1:1,000; Ser2448; Cell Signaling Technology, Danvers, MA, United States), and subsequently incubated with goat anti-rabbit IgG H&L (ab150077; 1:500; Alexa Fluor® 488; Abcam, Cambridge, MA, United States). Rabbit monoclonal antibody to glyceraldehyde 3-phosphate dehydrogenase (GAPDH; 5,174; 1:1,000; Cell Signaling Technology, Danvers, MA, United States) was used as the protein-loading control of total lysates. Finally, the PVDF membranes were visualized using a G-BOX image capture system (Syngene, Cambridge, MA, United States), and band intensities were quantified using ImageJ 1.0 software (NIH, Bethesda, MD, United States).

### Statistical analysis

All data are presented as the mean ± standard deviation. The Shapiro-Wilk test was employed to assess data normality. Data were normally distributed. Differences between groups were analyzed using One-way analysis of variance followed by Tukey’s *post hoc* test or *t-*test, performed with SPSS 20.0 software (IBM, Almonk, NY, United States). Visualization was conducted using GraphPad Prism 8.0.0 software (GraphPad, San Diego, CA, United States). Differences were considered significant with a value of *P* < 0.05.

## Results

### Extended OGD duration results in decreased LC astrocyte survival

**FIGURE 1:**
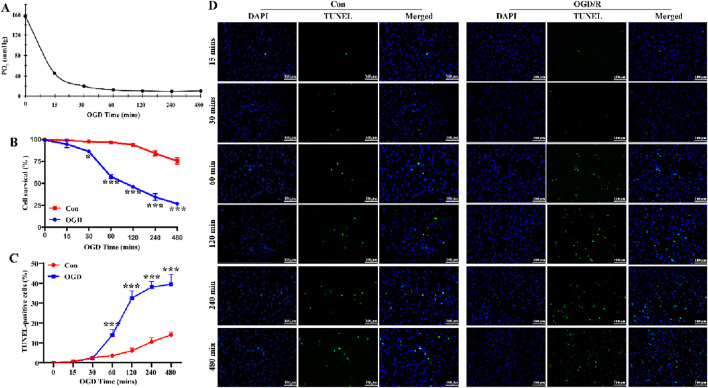
Extended OGD duration results in decreased LC astrocyte survival. **(A)** Partial pressure of oxygen (PO_2_) in the supernatant of cultured LC astrocytes, measured using the OxyLite. The partial PO_2_ was significantly reduced to below 10 mmHg, confirming that the cells were in a fully anoxic state. **(B)** Changes in LC astrocyte survival during OGD exposure, assessed using the CCK-8 assay. **(C,D)** TUNEL staining results showed that, compared with the Con group, OGD induced apoptosis in LC astrocytes. Scale bars: 100 μm ^*^ *p* < 0.05, ^**^ *p* < 0.01, ^***^ *p* < 0.001 vs. Con group (*t-*test). *N* (Biological repetition) = 3 per group.

To establish a stable OGD cell model, we measured the PO_2_ in the cell supernatant and conducted CCK-8 assay and TUNEL staining to determine the optimal OGD exposure time. Cells were exposed to OGD for six time periods: 15, 30, 60, 120, 240, and 480 min. As shown in Figure 1A, the PO_2_ in the supernatant was significantly reduced to below 10 mmHg after 1 h of OGD exposure, confirming that the cells were in a hypoxic state. These results demonstrated that OGD (1% O2, serum-free, and no sugar) exposure for more than 1 h successfully created a hypoxic environment in the culture medium. Compared with the Con group, LC astrocytes in the OGD group exhibited reduced survival rate and increased numbers of TUNEL-positive cells (Figures 1B–D, *P* < 0.05). Within 30 min of OGD, LC astrocyte viability decreased gradually, and after 1 h, cell viability decreased significantly, reaching 40%–60%, and by 8 h, cell viability decreased to 30% (Figure 1B). The apoptosis rate of LC astrocytes ranged from 40% to 50% at 1 h of OGD induction (Figures 1C,D). Therefore, we used the 1 h of OGD treatment in subsequent experiments, as it significantly reduced cell survival (53.00% ± 6.56%) and enhanced apoptosis (41.67% ± 8.50%).

**FIGURE 2:**
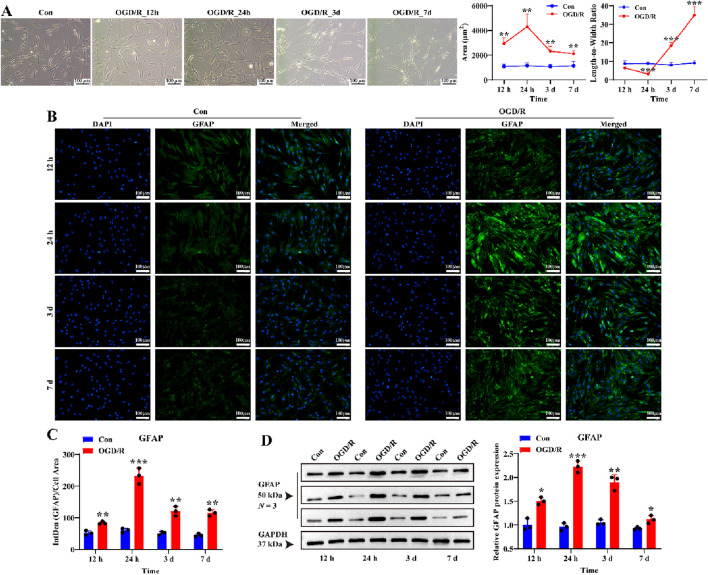
Effect of OGD/R on GFAP protein expression of LC astrocytes. **(A)** Morphological changes in LC astrocytes during OGD/R exposure. OGD/R at different durations resulted in pronounced changes in the area and length-to-width ratio of LC astrocytes. Scale bars: 100 μm. **(B,C)** Immunofluorescence staining showed that GFAP protein expression was significantly higher in the OGD/R group compared to the Con group. Scale bars: 100 μm. **(D)** Quantitative analysis revealed a significant increase in GFAP expression in the OGD/R group, with the highest expression observed in the OGD/R_24 h group. Quantitative analysis of GFAP expression was performed using Western blotting. Samples from the same experiment were processed in parallel for gels and blots. Mean fluorescence intensity was quantified based on the mean 8-bit pixel intensity values of the image. Mean fluorescence intensity is quantified by calculating the ratio of the integrated density (IntDen) of the target protein to the cell area, based on the average 8-bit pixel intensity value of the image. Protein expression levels were normalized to GAPDH expression. ^*^ *p* < 0.05, ^**^ *p* < 0.01, ^***^ *p* < 0.001 vs. Con group (*t-*test). *N* (Biological repetition) = 3 per group.

Next, we tested four reoxygenation time points (12 h, 24 h, 3 days, and 7 days) following 1 h of OGD to determine whether the OGD/R model could activate LC astrocytes. Following reoxygenation, the area of LC astrocytes increased significantly, especially at 24 h (Figure 2A). Additionally, OGD/R resulted in a pronounced decrease in the length-to-width ratio of LC astrocytes at 12 h and 24 h, and a pronounced increase at 3 days and 7 days (Figure 2A). Immunostaining and Western blotting revealed that GFAP expression was significantly elevated in LC astrocytes at 12 h, 24 h, 3 days and 7 days after recovery, peaking at 24 h post-reoxygenation (Figures 2B–D, *P* < 0.05). Thus, OGD/R induced the activation of LC astrocytes, promoting morphological changes and enhancing GFAP expression.

### Reactive LC astrocytes regulate VSMC morphology after OGD/R exposure

**FIGURE 3:**
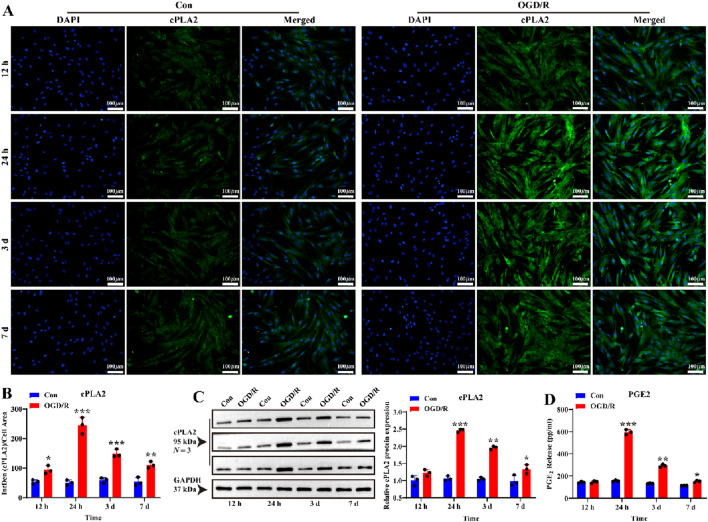
Effect of OGD/R on cPLA2 protein expression and PGE_2_ release of LC astrocytes. **(A,B)** Immunofluorescence staining revealed significantly higher cPLA2 protein expression in all OGD/R groups compared to, with the highest expression observed in OGD/R_24 h group. Scale bars: 100 μm. **(C)** Western blot analysis showed that the expression of cPLA2 protein was enhanced in all OGD/R group. Samples from the same experiment were processed in parallel for gels/blots. **(D)** PGE_2_ secretion was significantly upregulated in the OGD/R_24 h, OGD/R_3d, and OGD/R_7d groups compared to the Con group, with the highest secretion observed in the OGD/R_24 h group. Mean fluorescence intensity is quantified by calculating the ratio of the integrated density (IntDen) of the target protein to the cell area, based on the average 8-bit pixel intensity value of the image. ^*^ *p* < 0.05, ^**^ *p* < 0.01, ^***^ *p* < 0.001 vs. Con group (*t-*test). *N* (Biological repetition) = 3 per group.

To investigate whether reactive LC astrocytes play a protective role in blood vessel regulation, similar to cortical astrocytes (Wahis et al., 2021), we measured vasoactive arachidonic acid (AA) metabolites around the LC astrocytes. LC astrocytes and supernatants were collected after 1 h of OGD exposure and recovery for 12 h, 24 h, 3 days, and 7 d cPLA2 expression, which indicates AA release from cells, peaked after 24 h of recovery and slightly decreased after 7days (Figures 3A–C, *P* < 0.05). Notably, PGE_2_ protein secretion in the supernatant was increased after 24 h of recovery (Figure 3D, *P* < 0.05). These indicates that LC astrocytes secrete vasoactive factors following OGD/R exposure.

**FIGURE 4:**
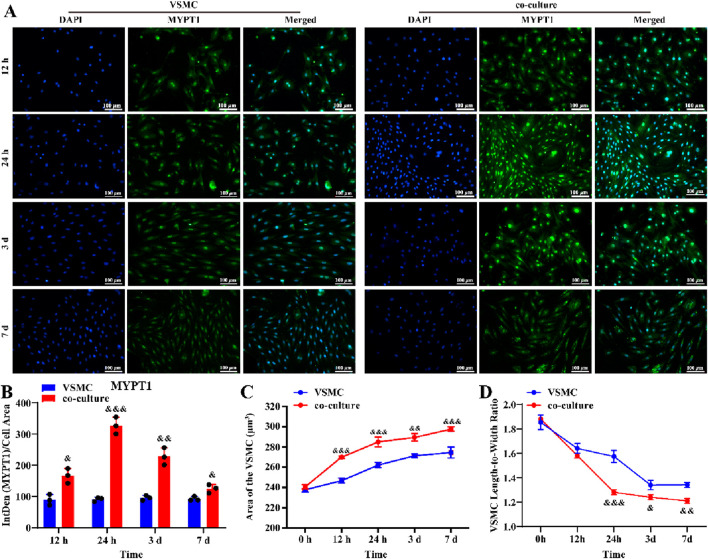
LC astrocytes contribute to the relaxation of co-cultured VSMCs under the OGD/R cultured. **(A,B)** Immunofluorescence staining revealed a significant upregulation of MYPT1 protein expression in LC astrocytes co-cultured with VSMCs, compared to VSMCs alone. **(C,D)** Compared with the VSMC group, the co-culture significantly enhanced the relaxation of VSMCs. Scale bars: 100 μm. Mean fluorescence intensity is quantified by calculating the ratio of the integrated density (IntDen) of the target protein to the cell area, based on the average 8-bit pixel intensity value of the image. ^&^ *p* < 0.05, ^&&^ *p* < 0.01, ^&&&^ *p* < 0.001 vs. VSMC group (*t-*test). *N* (Biological repetition) = 3 per group.

To further investigate this, we established a LC astrocyte-VSMCs co-culture system. Immunofluorescent staining revealed that MYPT1 expression was higher in VSMCs co-cultured with reactive LC astrocytes compared to those cultured alone (Figures 4A,B, *P* < 0.05). Compared to the Con group, the cell surface area of VSMCs was significantly larger, and the cell length-to-width ratio was significantly smaller in the co-culture group (Figures 4C,D, *P* < 0.05), suggesting a relaxed VSMC morphology. Taken together, these findings reveal that reactive LC astrocytes regulate VSMC morphology following OGD/R exposure.

### The mTOR signal pathway mediates the function of reactive LC astrocytes

**FIGURE 5:**
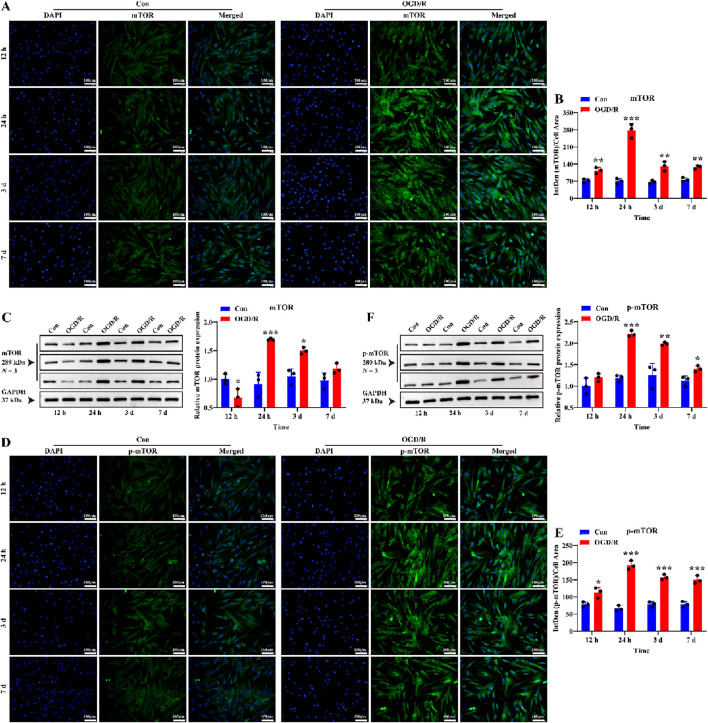
Effect of OGD/R on mTOR and p-mTOR protein expression of LC astrocytes. **(A,B)** Immunofluorescence staining revealed a significantly higher expression of mTOR protein in the OGD/R group compared to the Con. Scale bars: 100 μm. **(C)** Quantitative analysis by Western blotting showed enhanced mTOR protein expression in both the OGD/R_24 h and OGD/R_3d groups. **(D,E)** Representative images of p-mTOR immunofluorescence staining and statistical analysis of the mean fluorescence intensity of p-mTOR in each group. Scale bars: 100 μm. **(F)** The Quantitative analysis of p-mTOR expression by Western blotting. Mean fluorescence intensity is quantified by calculating the ratio of the integrated density (IntDen) of the target protein to the cell area, based on the average 8-bit pixel intensity value of the image. ^*^ *p* < 0.05, ^**^ *p* < 0.01, ^***^ *p* < 0.001 vs. Con group (*t-*test). *N* (Biological repetition) = 3 per group.

To further explore the mechanisms linking reactive LC astrocytes to vascular regulation, we hypothesized that the mTOR mediates their function. We first investigated whether mTOR was activated in LC astrocytes after 1 h of OGD/R. The results showed that the expression of mTOR and p-mTOR were significantly higher after recovery (Figures 5A–F, *P* < 0.05), demonstrating that reactivation of LC astrocytes following OGD/R was associated with activation of mTOR.

**FIGURE 6:**
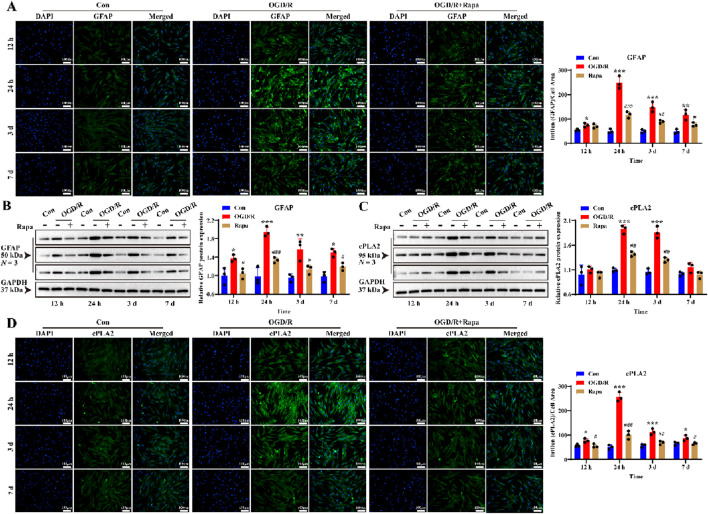
Effect of mTOR inhibitor on GFAP and cPLA2 protein expression of LC astrocytes during OGD/R. **(A,B)** The expression of GFAP and cPLA2 protein in the Rapa treatment group was significantly lower than in the OGD/R group, as assessed by **(A)** immunofluorescence staining and **(B)** Western blotting. Scale bars: 100 μm. **(C,D)** Quantitative analysis by **(C)** Western blotting and **(D)** immunofluorescence staining showed that the expression of cPLA2 protein was decreased in all Rapa treatment groups compared to the OGD/R group. Mean fluorescence intensity is quantified by calculating the ratio of the integrated density (IntDen) of the target protein to the cell area, based on the average 8-bit pixel intensity value of the image. ^*^ *p* < 0.05, ^**^ *p* < 0.01, ^***^ *p* < 0.001 vs. the Con group; ^#^ *p* < 0.05, ^##^ *p* < 0.01, ^###^ *p* < 0.001 vs. the OGD/R group (One-way analysis of variance and Tukey’s *post hoc* test). *N* (Biological repetition) = 3 per group.

To further investigate the role of mTOR in reactive LC astrocytes, we pre-treated primary LC astrocyte cultures with rapamycin prior to OGD/R exposure. A similar effect was observed for GFAP protein expression at 24 h and 3 days (Figures 6A,B, *P* < 0.05). These results suggest that rapamycin attenuated the increased levels of GFAP in LC astrocytes following OGD/R. We then assessed the expression of cPLA2 to further investigate the role of the mTOR pathway. Both immunofluorescence and Western blot analysis demonstrated that rapamycin treatment suppressed the changes in cPLA2 expression observed in LC astrocyte cultures following OGD/R (Figures 6C,D, *P* < 0.05). Therefore, mTOR signal pathway mediates the function of LC astrocytes in regulating cPLA2 levels.

## Discussion

Our findings indicate that LC astrocytes are activated after exposure to varying durations of OGD/R, thereby regulating VSMC morphology and modulating protein expression and PGE2 secretion. These results suggest that LC astrocytes may participate in the mechanisms underlying the autoregulatory function of ONH vessels.

Retinal optic nerve ischemia-reperfusion (I/R) injury is a common pathophysiological event in ischemic eye diseases, such as central retinal arteriovenous occlusion, diabetic retinopathy, glaucoma. It involves the interaction of various functional cells, cytokines, transcription factors, and molecular pathways. *In vitro* OGD/R is commonly used to replicate I/R conditions. However, the optimal time and environmental conditions for inducing OGD/R in astrocytes remain a topic of debate. We refined the OGD/R method for astrocytes by integrating previous experimental findings and literature. Some studies have employed anaerobic incubators (95% N_2_, 5% CO_2_) or simple anaerobic generators (0% O_2_) to establish the OGD model. However, we found that anaerobic cultivation often creates an acidic environment, which not only distorts the OGD/R results but also impedes subsequent reperfusion experiments due to inadequate activation. To ensure the OGD environment, we utilized a triple-gas incubator (1% O_2_, 5% CO_2_, and 94% N_2_) and a tissue monitoring system to measure PO_2_ levels in the culture supernatant. Additionally, we employed CCK-8 and TUNEL assays to determine the optimal OGD duration. Our results indicated that the optimal duration of OGD for LC astrocytes is 1 h.

Astrocytes are considered important for supporting and maintaining the homeostasis within the central nervous system and retinal ganglion cells (Shinozaki and Koizumi, 2021; Escartin et al., 2021). Activated astrocytes are vigorously involved in neuronal synapse formation and protect nerve tissues by releasing growth factors or cytokines, remodeling the extracellular matrix and forming the glial scar (Bon et al., 2021). In our study, following exposure to OGD/R, activated LC astrocytes exhibited characteristic changes in cell morphology and upregulation of GFAP expression. Notably, after reoxygenation (especially 12 h and 24 h), the cell area of LC astrocytes increased, while the length-to-width ratio decreased; however, at longer reoxygenation time points (3 days and 7 days), the length-to-width ratio increased instead. This biphasic pattern of morphological changes suggests that early LC astrocytes (12–24 h of reoxygenation) may undergo rapid reactive hypertrophy and cell body expansion, whereas at later stages LC astrocytes (3-7 days of reoxygenation) may be involved in more complex remodeling processes (Panickar and Norenberg, 2005; Yu et al., 2021; Li et al., 2019; Shigetomi et al., 2019).

Notably, the time course of the activation of astrocyte varied across studies (Sun et al., 2013; Coudrillier et al., 2012). In our study, LC astrocytes were activated after 1 h of OGD followed by recovery for 12 h, 24 h or 3 days. The morphological changes in activated LC astrocytes peaked after 24 h of recovery. The selection of reoxygenation time points and the observed peak response at 24 h warrant further contextualization within *in vivo* pathophysiology. Notably, GFAP-labeled astrocytes peaked at 24 h in a mouse model of retinal ischemia-reperfusion (Hu et al., 2022), which is consistent with the phenomenon observed *in vitro*. A similar situation has been observed in animal models of glaucoma and ocular hypertension (Oh et al., 2024; Ling et al., 2020). Therefore, the OGD/R model of the present study mimics to the activation state of LC astrocytes in the *in vivo* model. In contrast, Sun et al. (2013) reported the most prominent changes in activated astrocytes 3 days after elevating intraocular pressure and recovery, with a return to the resting state between 1 and 6 weeks. Coudrillier et al. (2012) reported that astrocytes become activated at the first day after mechanical injury to the ONH, peaking at 3 weeks. Several factors may account for these differences. One likely reason is the differing injury paradigms employed in these studies. The studies by Sun et al. (2013) and Qu J et al. (Coudrillier et al., 2012) did not induce ischemia; Sun et al. increased intraocular pressure, while Qu J et al. caused mechanical damage. Mechanical (or stress) injury induces continuous and progressive inflammatory and toxic effects, potentially leading to prolonged the activation of astrocytes (García-Bermúdez et al., 2021). In contrast, our OGD/R model focused on ischemia, which may promote nerve recovery and repair. Therefore, the activation response is rapid and direct. We hypothesize that distinct influencing factors activate different subtypes of astrocytes, resulting in varying responses and functions (Fan and Huo, 2021). Another potential explanation is the use of different species in these studies. In the studies by Sun et al. and Qu J et al., the authors focused on fibrous LC astrocytes in mice. In contrast, we studied rat LC protoplasmic astrocytes, which exhibit functional similarities relevant to vascular autoregulation and shape with human LC astrocytes (Strickland et al., 2022; Tan et al., 2018; Lopez et al., 2020; Oberheim et al., 2009). The early, rapid morphological remodeling we observed underscores the ability of LC astrocytes to respond rapidly and potentially participate in early vasoregulatory or neuroprotective processes in the context of ischemic injury. This is similar to the known role of astrocytes in rapid response in acute cerebral ischemia (Lee et al., 2022; Giovannoni and Quintana, 2020; Hasel and Liddelow, 2021).

The neurovascular unit consists neuronal cells, astrocytes, and vascular wall cells (smooth muscle cells, endothelial cells) and forms an integral component of the blood-brain barrier or blood-retinal barrier (Rudraraju et al., 2020; Yang et al., 2020). Our study demonstrated that the activation of LC astrocytes by OGD/R *in vitro* significantly increased cPLA2 protein expression and PGE2 secretion into the cell supernatant. Furthermore, Activated LC astrocytes lead to an increase in MYPT1 expression and area and a decrease in length-to-width ratio of VSMCs. Notably, the expression level and phosphorylation of MYPT1 directly determines myosin phosphatase (MLCP) activity, thereby precisely controlling the phosphorylation level of MLC20, a “molecular switch” that ultimately determines whether VSMCs are diastolic, contractile, or maintain a specific muscle tone (Turner and Macdonald, 2014; Butler et al., 2013; Ogut and Brozovich, 2008). This suggests that LC astrocytes may regulate contraction/relaxation and muscle tone of local blood vessels by mediating MYTP1 expression in VSMCs. Recent studies corroborate our findings (Drew, 2019).

The mTOR pathway, regarded as the “metabolic control center” of eukaryotic cells, widely operates in the nervous system in the development of axons, dendrites, and synapses (Zou et al., 2020). Previous studies have demonstrated that the mTOR pathway is associated with the activation of astrocytes, phenotypic switch in VSMC, and the expression of cPLA2 and PGE_2_ (Querfurth and Lee, 2021; Wen et al., 2013; Li et al., 2018; Yang and Zhang, 2018; Villa-González et al., 2022; Han et al., 2021). Therefore, the present study investigated the changes in the activity of mTOR pathway in LC astrocytes under OGD/R conditions. In our study, we found that the expression of mTOR and p-mTOR was enhanced in OGD/R-activated LC astrocytes, with temporal changes in expression paralleling those observed for GFAP and cPLA2. Furthermore, treatment with the mTOR inhibitor rapamycin suppressed changes induced by the activation of LC astrocytes, indicating that OGD/R activates LC astrocytes though the mTOR pathway. Our findings align with previous reports (Villa-González et al., 2022). The cPLA2-PGE_2_ axis is one of the central drivers of the transition of astrocytes from a resting state to a pro-inflammatory, reactive state (Sun et al., 2005; Sun et al., 2014; Stephenson et al., 1999). In astrocyte-mediated vascular remodeling, cPLA2 is the key initiator, which drives the synthesis of PGE_2_ through the release of AA. PGE_2_ mediates vasodilation, increases vascular permeability, amplifies inflammatory responses, and promotes angiogenesis through its receptors (EP2/EP4) (Sun et al., 2005; Sun et al., 2014; Stephenson et al., 1999). In this study, a series of changes in astrocytes and VSMCs corresponded to the function of the cPLA2-PGE_2_ axis. Previous studies have demonstrated that cPLA2 is a potent intracellular stimulator of mTOR, a process that is dependent on the Rheb and PI3K/Akt pathways (Wen et al., 2013; Li et al., 2018; Yang and Zhang, 2018). Therefore, cPLA2 not only regulates LC astrocyte-mediated vascular remodeling but is also associated with mTOR pathway-mediated LC astrocyte activation.

Notably, our study has several limitations. Although we confirmed extracellular hypoxia via PO_2_ monitoring and functional cellular responses, future studies could incorporate direct cellular hypoxia detection assay to further validate the model. GFAP is a useful but insufficient marker for astrocytes (Escartin et al., 2021). Therefore, detection of ALDH1L1, ALDOC, GS, and proliferation markers (PCNA and Ki67) in *in vivo* and *in vitro* samples is needed to further support the findings of this study. The heterogeneity of astrocytes between LC and other regions needs to be characterized by means of single-cell sequencing and transcriptomics, as in previous studies (Cahoy et al., 2008; Gao et al., 2023; Matusova et al., 2023; Zamanian et al., 2012). Moreover, whether the effects of LC astrocytes on VSMC morphology, contraction/relaxation, and muscle tone are related to MYTP1 remains to be verified in the future by gene editing and inhibitors/activators. Importantly, the effects of LC astrocytes on the morphology of VSMCs and ONH vascular remodeling through the cPLA2-PGE2 and mTOR pathways remain to be validated in animal models and LC astrocyte/VSMC co-culture system.

## Conclusion

We demonstrated that OGD/R conditions *in vitro* activate LC astrocytes through the mTOR pathway. Activated LC astrocytes exhibited increased expression of GFAP and cPLA2 proteins, as well as enhanced secretion of PGE_2_, thereby regulating VSMC dilation. Therefore, LC astrocytes may regulate the local blood flow and contribute to the mechanisms underlying the autoregulatory function of ONH vessels. This finding may enhance our understand of LC astrocytes better. Additionally, it provided a novel perspective for further investigating the etiology and exploring treatment options for eye diseases related to optic nerve fiber ischemic injury.

## Data Availability

The raw data supporting the conclusions of this article will be made available by the authors, without undue reservation.
